# “What If We Get Sick?”: Spanish Adaptation and Validation of the Fear of Illness and Virus Evaluation Scale in a Non-clinical Sample Exposed to the COVID-19 Pandemic

**DOI:** 10.3389/fpsyg.2021.590283

**Published:** 2021-03-11

**Authors:** Marianne Cottin, Cristóbal Hernández, Catalina Núñez, Nicolás Labbé, Yamil Quevedo, Antonella Davanzo, Alex Behn

**Affiliations:** ^1^Facultad de Medicina, Universidad de Chile, Santiago, Chile; ^2^Escuela de Psicología, Universidad Finis Terrae, Santiago, Chile; ^3^Instituto Milenio para la Investigación en Depresión y Personalidad, MIDAP, Santiago, Chile; ^4^Escuela de Psicología, Universidad Adolfo Ibáñez, Santiago, Chile; ^5^Facultad de Ciencias Sociales, Escuela de Psicología, Pontificia Universidad Católica de Chile, Santiago, Chile; ^6^Departamento de Psiquiatría y Salud Mental Oriente, Facultad de Medicina, Universidad de Chile, Santiago, Chile

**Keywords:** COVID-19, FIVE, fear, Spanish adaptation and validation, pandemic, psychological impact, virus, SARS-CoV-2

## Abstract

Distinct sources of stress have emerged during the COVID-19 pandemic. Particularly, fear is expected to generate significant psychological burden on individuals and influence on either unsafe behavior that may hinder recovery efforts or virus-mitigating behaviors. However, little is known about the properties of measures to capture them in research and clinical settings. To resolve this gap, we evaluated the psychometric properties of a novel measure of fear of illness and viruses and tested its predictive value for future development of distress. We extracted a random sample of 450 Chilean adult participants from a large cross-sectional survey panel and invited to participate in this intensive longitudinal study for 35 days. Of these, 163 ended up enrolling in the study after the demanding nature of the measurement schedule was clearly explained to them. For this final sample, we calculated different Confirmatory Factor Analyses (CFA) to evaluate the preliminary proposed structure for the instrument. Complementarily, we conducted a content analysis of the items to qualitatively extract its latent structure, which was also subject to empirical test via CFA. Results indicated that the original structure did not fit the data well; however, the new proposed structure based on the content analysis did. Overall, the modified instrument showed good reliability through all subscales both by its internal consistency with Cronbach’s alphas ranging from 0.814 to 0.913, and with test–retest correlations ranging from 0.715 to 0.804. Regarding its convergent validity, individuals who scored higher in fears tended to also score higher in depressive and posttraumatic stress symptoms at baseline. Furthermore, higher fears at baseline predicted a higher score in posttraumatic stress symptomatology 7 days later. These results provide evidence for the validity, reliability, and predictive performance of the scale. As the scale is free and multidimensional potentially not circumscribed to COVID-19, it might work as a step toward understanding the psychological impact of current and future pandemics, or further life-threatening health situations of similar characteristics. Limitations, practical implications, and future directions for research are discussed.

## Introduction

17,334,539 is the number of people who, as of July 31th 2020, have been identified worldwide as positive cases of Coronavirus 2019 (COVID-19). This disease, declared 6 months ago as a public health emergency of international concern, is caused by the SARS-CoV-2 virus consisting of a new human pathogen with a high transmission capacity with animals (Rodríguez-Morales et al., 2020). Of the total infected, 674,038 people have died (World Health Organization, 2020). The first number (of cases) is equivalent to the total population of countries, such as the Netherlands or Syria, while the second number is higher than the total population of Luxembourg.

A disease is declared a pandemic when the transmission between people without immunity surpasses what was expected on a global scale (Morens et al., 2009). Furthermore, the virus has appeared in the age of technology. This has permitted us to witness the impressive pace at which the number of cases is rising and re-surging in waves across countries. This information can be obtained through official reports issued in real time by the World Health Organization through ProMEDmail or by “Coronavirus COVID-19 Global Cases/CSSE,” a specialized website of Johns Hopkins University (Bonilla-Aldana et al., 2020; Dong et al., 2020).

The coronavirus arrived in Latin America on the 26th of February when the first case of infection was confirmed in Brazil (Rodríguez-Morales et al., 2020). Since then, it has spread rapidly, encountering a 640,000,000-population region ill-prepared for this massive sanitary challenge and the social, economic, and psychological ramifications of the crisis (Biscayart et al., 2020). To reduce the viral transmission, each country in the South American region has scrambled to activate mitigation measures, including recommended confinement in some countries and mandatory lockdowns in others (Rodríguez-Morales et al., 2020). In practical terms, the strategy has been to isolate those who are infected, quarantine those who have potentially been exposed to the disease, and keep social contact to a minimum (Brooks et al., 2020), with extra precautions on high-risk populations such as the elderly and people with previous illnesses (Public Health England, 2020). Despite these strategies, by the time this paper is being written, three South American countries belong to the top 10 list with the highest number of confirmed cases (2,610,102 in Brazil; 400, 683 in Perú; and 353, 536 in Chile), the United States being the highest globally (4,496, 737) where 17% of the population is Hispanic or Latin American.

Chile, in particular, has been a highly affected country with 355.667 and 9.457 deaths. Moreover, we believe fear reports will be especially variable within the country considering the differential strategy that the Chilean government implemented compared to the rest of the continent. Most of Latin American countries implemented expansive quarantives and national lockdowns, while Chile started with partial lockdowns in specific districts, starting with the ones where COVID-19 was supposedly originated due to people returning from holidays outside the country.

Even though scholars and researchers of all disciplines have invested time and effort outlining tentative approaches to make predictions, it is still difficult to estimate the impact that this pandemic will have on our way of conceiving the world and our way of living. There are traditional epidemiological tools, but since it is a new virus, with unknown characteristics, it is more complicated to make predictions using the trajectory of previous diseases (The Lancet, 2019). Because of this, the balance between the benefit of such unpleasant but necessary coping strategies needs to be weighed against the present and future costs (Brooks et al., 2020).

Uncertainty, loneliness, vulnerability, economic insecurity, fear of infection, and facing death for ourselves or our loved ones are among the distinct sources of stress that have emerged in the pandemic’s setting and are expected to generate a significant burden on individuals (Lima et al., 2020; Montemurro, 2020; Moreno et al., 2020; Thakur and Jain, 2020). Gu et al. (2015) found that after the H1N1 epidemic, 45% of those surveyed felt fear for themselves or a loved one to become infected, and around 10% felt panic because of the contagion. Unconfirmed beliefs about the virus and perceived lethality increased emotional affectation, which underscores the value of providing adequate information from reliable sources (Gu et al., 2015).

In the setting of the current pandemic, though heterogeneous, preliminary evidence obtained mostly through online surveys reveals moderate-to-severe impact on the mental health of the general population, including an increase in symptoms of depression and posttraumatic stress disorders (Moreno et al., 2020; Tang et al., 2020). This impact seems to be higher in younger people (Tang et al., 2020) and at-risk populations, including individuals with preexisting mental health problems (Steenblock et al., 2020), who have or have been infected (Bo et al., 2020) or who are health-care workers (Chen Q. et al., 2020).

Understanding the scope and intensity that the COVID-19 pandemic has on mental health requires appropriate instrumentation to track directly related stressors. In addition to standard measurements of psychopathology (e.g., depression, anxiety, trauma), the unprecedented nature of this emergency likely requires the development of new instrumentation covering constructs directly related to the virus.

### The Importance of Measuring Fear Specifically Related to COVID-19

The construct of fear, as related to the COVID-19 pandemic, requires attention. Fear, as one of the basic emotions of the human being (Plutchik, 1980), plays a fundamental role in survival and adaptation to the environment, since it fulfills two important functions: first, the activation of physiological systems that prepare the organism for flight and defense, and secondly, avoiding exposure to potentially harmful stimuli through learning and cognitive assessment of danger or threat (Sosa and Capafóns, 2003; de Hoog et al., 2008). Although fear is functional and adaptive for humans, it ceases to be so when it occurs in the absence of a real danger or when it appears excessively, becoming a complex problem that is difficult to control (Martínez Pérez et al., 2009). Knowing the different fear levels might help us develop specific programs for groups of people according to certain characteristics or identify which of them require specific considerations because of a particular risk (Pakpour and Griffiths, 2020).

In the setting of natural disasters or traumatic events, the fear intensity seems to have a bearing on the development or exacerbation of mental health problems, appearing to be particularly relevant in the context of a virus as reported in previous pandemics (Maunder et al., 2003), as well as for COVID-19 (Moreno et al., 2020; Tang et al., 2020). In a sample of 2,485 participants, Tang et al. (2020) found that feeling extreme fear was one of the most significant predictors of both depression and posttraumatic stress disorder (PTSD), which may have higher and more lasting implications than the actual pandemic (Ornell et al., 2020).

The still unknown short- and long-term medical consequences of contracting COVID-19, with the invisible and rapid airborne dissemination mechanism, naturally comes with uncertainty and major changes in lifestyle that can further promote a sense of fear in the population (Huang and Zhao, 2020). In particular, fears of infection can promote stigmatization and discrimination against infected individuals or those thought to be infected, leading to increased depression, anxiety, and even suicide. The latter was anecdotally reported by Mamun and Griffiths (2020) in their account of the first suicide related to COVID-19 fear and more systematically by Dsouza et al. (2020) in India.

Also, above and beyond adverse mental health outcomes related to individual fears, “pandemic fear” may hinder recovery efforts, promote unsafe behaviors, and inhibit prosocial behaviors (e.g., hoarding, violence against health professionals, discrimination against potentially infected individuals, and stigma toward the certain societies) (Ahorsu et al., 2020; Lin, 2020). Most of these may be prevented since they seem to be consequences of misperception and misinformation among individuals, particularly when recommendations are constantly changing (Lin, 2020).

Interestingly, virus-mitigating behaviors, such as compliance with recommendations and enforced indications to prevent infection, have also been related to fear of COVID-19, specifically “functional fear.” This is thought to promote compliance with ordinances and thus decrease infection rates. In an international study, the fear of COVID-19 was the only significant predictor of several virus-mitigating behaviors, including hand washing and social distancing (Harper et al., 2020; Pakpour and Griffiths, 2020).

Taken together, all this evidence points to the importance of measuring fear as specifically related to COVID-19.

### Scales Developed for Measuring Fear During the COVID-19 Pandemic

Some measures have been developed for this purpose since the beginning of the pandemic, including the Fear of COVID-19 Scale (FCV-19S Ahorsu et al., 2020) and the Fear of Illness and Virus Evaluation (FIVE, Ehrenreich-May, in preparation). The efforts for developing appropriate scales might help advance research on the specific contribution of fear to the development of mental health problems as well as to individual behaviors related to community-level recovery (e.g., less stigmatization, pro-social and virus-mitigating behaviors).

On one hand, the FCV-19S is a very short seven-item, unidimensional measure used to measure the fear level of COVID-19. This has the advantage of being easier to understand and truly capture the essence of the fear construct. We believe that this scale is appropriate when the goal is to measure severity of fear, since it has one dimension and robust psychometric properties. This scale seems to be very useful for massive-scale studies and for comparing intersubject levels of fear.

Compared to the FCV-19, the FIVE is a multidimensional measure that, even though it is longer, may have the potential to disaggregate different dimensions related to fear. We believe that it would be useful to develop and validate scales that could let us comprehend the different motives behind one to be fearful about COVID-19, particularly in such uncertain context. Distinct facets, as measured by the FIVE, may be differentially related to these outcomes allowing grouping specific information about fear and thus more nuanced associations with other psychological and behavioral outcomes. This may help us understand community-level responses and develop adapted and contingent strategies.

### The Fear of Illness and Virus Evaluation

The FIVE (Ehrenreich-May, in preparation) was developed in this context, to measure specific fears related to the possibility of infection and the socioemotional distress caused by it. The original scale is freely available in the Supplementary Materials section. It may be freely used with permission from Jill Ehrenreich-May, Ph.D. (j.ehrenreich@miami.edu). As identified in the literature, fears of illness can be a moderator for the impact of the pandemic on mental health (i.e., more fears, more psychological vulnerability) (Moreno et al., 2020; Tang et al., 2020). The FIVE is an original self-report questionnaire for adults that measures fears of illness and viruses across four dimensions: (1) fears associated with infection and illness, (2) fears associated with social distancing, (3) behaviors associated with fear of illness and the virus, and (3) the functional impact of fears associated with illness and the virus. The first two dimensions are directly related to fears whereas the third one relates to behaviors due to the fears and the fourth dimension presents an overall measure of functional affectation. The questionnaire has 35 Likert-scale type items. It was originally developed in English, and to date, there are no published validations in Spanish and psychometric properties of the original measure (including internal consistency, factor structure, dimensionality analysis, test–retest reliability, and convergent validity) have not been published yet.

Even though there are other, specifically developed measures of COVID-19 fears available, the FIVE has two potential advantages. First, it has a theoretical multidimensional structure that may allow for the disaggregation of specific information about the construct and thus a more nuanced examination of the relationships between fear of illness and mental health or behavioral outcomes. Secondly, it is a more general measure of fear of illness and virus, which means it can be used in different situations where illness may be the source of fear for individuals. In other words, it has a larger generalization for its current and future use.

## Materials and Methods

### Participants

Data was collected between April and June 2020 and coincided with general social distancing recommendations and a mandatory quarantine due to the COVID-19 pandemic in several provinces of Chile. In the study, 450 participants were purposely sampled (Campbell et al., 2020) from a pool of 2,757 voluntary participants initially contacted through social networks who signed consents and provided basic socio-demographic information. This reduction of the sample was conducted in order to correct for typical biases of online convenience sampling, including a majority of female participants (88.6%) and a disproportionate number of students and younger participants (28.4%). Because of these biases, concerns have been raised about the use of convenience sampling and online surveys during the COVID-19 pandemic (Pierce et al., 2020). We attempted to correct for these biases by using quota sampling procedures, and the random sample of 450 participants was forced to maintain a 50% female–male ratio and a cap of 9% of students, which is in line with known population parameters in Chile. An additional reduction in the sample was observed when the demanding nature of the study protocol (i.e., daily prompts for 35 days and subsequent follow-ups at 2, 4, and 6 months) was clearly explained to the invited participants. At this stage, of the 450 invited participants, only 163 participants registered and downloaded the study app. Even though sample size was significantly smaller than expected, this was the result of a trade-off between a larger, unbalanced sample and a smaller, more committed sample, balanced for gender and proportion of students.

Table 1 shows the demographic and clinical characteristics of the final sample (*N* = 163). When available, reliable population benchmarks are presented to describe the extent to which the study sample differs from population parameters. It can be seen that in terms of age, gender, and work status distribution the study sample is similar to the population. However, it is also evident that the study sample differs significantly from population parameters related to psychosocial factors, including education and income. The comparison between the study sample and population estimates for clinical variables is also of interest. The depression benchmark indicates the prevalence of depressive symptomatology (i.e., root symptoms of anhedonia and low mood) in the general population. As expected, in a sample of adults exposed to the COVID-19 pandemic and related restrictions and alterations of daily life, depressive symptomatology is higher, even using a more stringent criterion (PHQ-9 > 10) (Margozzini and Passi, 2018). The PTSD benchmark is interesting because it comes from a population-representative study that used a short posttraumatic symptomatology screener (PTSS) just after the Chilean 2010 earthquake and tsunami. The percentage of individuals presenting with these symptoms after the earthquake and tsunami is similar to the estimate of individuals with PTSD in our study sample. Of note, in coastal areas most affected by the earthquake and the tsunami, the percentage of individuals endorsing post-traumatic symptoms rises to 13.1% (Abeldaño et al., 2014).

**TABLE 1:** Demographic characteristics of the participants.

Sociodemographic variables	Study sample (*N* = 170)	Population benchmarks
Age	32.21 (9.32)	35.8
Female	60.6%	51.1%^a^
**Education (highest achieved)**		
Elementary	0%	25.6%*^a^
High school	16%	44.6%*^a^
Technical degree	10.7%	
Professional (university degree)	54.4%	29.8%*^a^
Postgraduate	18.9%	
**Work status**		
Currently unemployed	17.8%	11.2%^a^
Homemaker	4.1%	
Student	11.2%	
Independent worker	20.1%	23%^b^
Dependent worker	45.6%	47.6%^a^
Retired	1.2%	
**Lives with:**		
Couple	17.1%	12.7%^a^
Couple and children	29.4%	28.8%^a^
Parents	31.2%	
Other family members	27.1%	19.0%^a^
Alone	7.7%	17.8%^a^
Alone with children	4.1%	12.7%^a^
Median family income in CLP	810.000	787.000^a^
**Clinical variables**		
Participants with moderate to severe depression at baseline (>10 in PHQ-9)	53.4%	15.8%^c^
Participants with PTSD at baseline (ITQ)	13%	11.3%^d^
Average FIVE score (represents percentage from 0 to 100%)		
Fears of getting sick	34.4%	
Fears that others might get sick	36.4%	
Fears of concrete limitations	31.2%	
Fears of not being able to meet the basic needs	39.1%	

^a^Apablaza and Vega, 2018. ^b^Instituto Nacional de Estadísticas [INE], 2018. ^c^Margozzini and Passi, 2018. ^d^These values correspond to the prevalence of PTSD in a sample exposed to a major earthquake in 2010 (Diaz Silva, 2011; Zubizarreta et al., 2013). *Educational level reached at 25 years of age.

### Procedure

The 163 participants who were selected from the initial large sample were invited to participate in the intensive phase of the study, which lasted 35 days. All the measurements taken during the study were collected from the participants’ cell phones through a commercially available application (Ethica Data Services Inc, 2020), who provided us with a free license for this study. The participants received instructions to download the application, through which questionnaires and questions were sent to the participants’ smartphones (Android or IOS).

After registering for the study and installing the application, participants completed a more detailed socio-demographic characterization instrument, with a baseline consisting of the Patient Health Questionnaire-9 (PHQ-9), the International Trauma Questionnaire (ITQ), and the FIVE. We re-sent these instruments for participants to complete every 5 (PHQ-9) or 7 days (ITQ and FIVE) during the 35 days of the intensive phase of the study. During the study, participants also completed daily measurements of positive and negative emotionality, satisfaction with romantic relationships, parenting roles, and social support, which were part of the broader study. There was also daily monitoring of hours of sleep, fluctuations in appetite, weight, substance use, daily hours of exercise, contact on social media through the internet, and internet use. Lastly, we collected passive information from their cellular device registered through pedometry, ambient light, GPS location, and screen time.

During the intensive phase of the study, we held two raffles with prizes to compensate participants.

The study protocol was authorized by the Committee of Ethics in Science of Universidad Adolfo Ibáñez, prior to data collection.

### Measures

#### Fear of Illness and Virus

We used the Spanish version of the Fear of Illness. This version is available in the Supplementary Materials section. We used the “Fear of Illness and Virus Evaluation” (FIVE) (Ehrenreich-May, in preparation), a scale designed during the current COVID-19 pandemic to evaluate fear and fear-associated behaviors. The adult version was originally designed in English and consists of 35 items on a 4-point Likert scale. Its first 19-item range from “I’m not afraid of this at all” to “I’m afraid of this all the time,” reflecting two dimensions: “Fears about Contamination and Illness” (items 1–9) and “Fears about Social Distancing” (items 10–19). The instrument also includes a third part consisting of a list of potential behaviors related to the previously mentioned fears (e.g., staying away from people and using hand sanitizer), ranging from “I have not done this in the last week” to “I did this all the time last week” (items 20–33). Finally, a fourth part has two questions about the impact of Illness and virus fears (experiencing strong emotions and getting in the way of enjoying life), ranging from “Not true for me at all” to “Definitely true” (items 34 and 35). To date, no psychometric properties have been reported for this instrument, while the author describes the organization of the items as “provisional subscales.”

#### Posttraumatic Stress Disorder and Complex Posttraumatic Stress Disorder

Posttraumatic stress was evaluated using the ITQ (Cloitre et al., 2018), based on the International Classification of Diseases 11th Revision (ICD-11) criteria for PTSD and complex posttraumatic stress disorder (CPTSD). It consists of 18 items on a 5-point Likert scale from 0 “Not at all” to 4 “Extremely.” The participant needs to identify a particularly troublesome experience, its onset, and the time frame in which it occurred, to then answer all questions in regard to this experience. The first six items measure the criteria for PTSD, while items 7–9 measure functional impairment related to it. Items 10–15 measure the criteria for CPTSD, while items 16–18 measure their related functional impairment. From that 12 items, specific items are then used to identify PTSD and CPTSD, and the 6 remaining represent the general functional impairment. The scale has been used dimensionally and showed good levels of general internal consistency (Cronbach’s α = 0.90; Ho et al., 2019). An available Chilean translation was used in the current study (Fresno et al., in preparation).

#### Depressive Symptomatology

Depressive symptomatology was measured using the nine-item “Patient Health Questionnaire” (PHQ-9; Kroenke et al., 2001), based on DSM-IV criteria (American Psychiatric Association, 1990). It consists of nine items on a 4-point Likert scale ranging from 0 “Never” to 3 “Almost every day,” measuring frequency of criteria met for the last 2 weeks. A version validated in Chile was used for the present study (Tomas Baader et al., 2012), which showed good reliability (Cronbach’s α = 0.835).

### Analytic Plan

We analyzed data using the statistical environment R (R Core Team, 2020) and Mplus 8 (Muthén and Muthén, 2017). We present descriptive statistics and a correlation matrix for the first measurement. Then, we present the computed set of CFA where we tested the underlying structure of the FIVE using the R library “lavaan” (Rosseel, 2012). We used a robust maximum likelihood (MLR) method of estimation to compensate for violations of the multivariate normality assumption (Kline, 2015). MLR corrects both standard errors and chi-square statistics for deviations from normality (Li, 2015). We assessed fit to the data using the more stringent criteria proposed by Hu and Bentler (1999; TLI > 0.95 CFI > 0.95, RMSEA < 0.06, and RMSR < 0.08).

We performed CFA to test six potential structures. First, we tested a one-factor structure where all items load in one general factor including items 20–35. Then, we computed a one “fear” factor excluding items 20–35. While this solution was not informed by the authors’ scoring instructions, we nonetheless examined it as a baseline comparison. We then tested a third factor using the four theoretical dimensions derived from the proposed provisional subscales described above (Ehrenreich-May, in preparation). Fourth, we computed a bi-factor model (Reise et al., 2010) in which a general factor captured the commonality between items, while four orthogonal factors capture residual variability not accounted for by the former.

Given the preliminary nature of the structures, we also made a content analysis of the items to propose latent dimensions based on the qualitative commonalities between the items and following an open coding technique based on the framework of grounded theory (Strauss and Corbin, 1998). We tested the proposed content-based structure with both its structure and a bi-factor approach.

After we calculated the CFAs and selected a final structure, we provide its internal consistency (Cronbach’s α, composite reliability or Omega, Average Variance Extracted, and item-total correlations), test–retest reliability (Pearson’s r and Intra-Class Correlations between time point 1 and the next), and convergent validity with depressive (PHQ-9), and posttraumatic stress symptomatology (ITQ). Finally, we assessed the instrument’s predictive validity by predicting depressive and posttraumatic symptomatology in a subsequent measurement wave, controlled by baseline levels with an OLS regression.

For both CFA and OLS regressions, missing values were handled by a listwise deletion method. For the regression analyses, sums were calculated for the rows with complete cases within a scale only.

## Results

Almost all correlations between the 19 first items were significant and positive. On the other hand, no clear correlation pattern appeared between items 20 and 33 (behaviors related to fears) and items 1–19 (fears). There was also no clear pattern within the behaviors. This may be an indication of two distinct sets of instruments, with the first 19 composing a scale of fears and the next 20–33 composing a set of individual behaviors. Most of the fear items were also correlated with the “impact” items (33–34) and with depressive and posttraumatic stress symptomatology, which was not evident for all distinct behaviors. The correlation matrix together with mean and standard deviations of the described variables are in Supplementary Materials.

### Initial Confirmatory Factor Analysis of the Original Theoretical Structure

As can be seen in the upper part of Table 2, none of the first latent structures derived from the provisional subscales fitted the data well. It seems that neither a general factor of fear, a composed factor structure including fears and behaviors, or a bifactor model accounted for the data’s latent structure. Because of this, the next step was to subject the instrument to content analysis.

**TABLE 2:** New four-factor solution loadings.

Item	Fears of getting sick	Fears that others may get sick	Fears of concrete limitations	Fears of not being able to meet the basic needs
1	0.772** (0.774)			
2	0.845** (0.853)			
3	0.789** (0.782)			
4	0.801** (0.797)			
5		0.467** (0.452)		
6		0.824** (0.847)		
7		0.618** (0.624)		
8		0.713** (0.695)		
9		0.479** (0.469)		
10			0.702**	
11			0.807**	
12				0.762**
13			0.715**	
14				0.584**
15			0.617**	
16			0.720**	
17			0.653**	
18				0.670**
19				0.796**

All loadings are standardized. **Significant at p < 0.01. Parentheses indicate the two fear factors solution loadings.

### Content Analysis, Development, and Testing of a Newly Proposed Factor Structure

Based on the low fit indices for the originally proposed factor structure, we set out to explore alternative distributions of items based on content analysis. Because the FIVE is largely based on substantive theory and not yet empirically derived, this can be considered a necessary step in the development of a strong measure. Looking at the items of the FIVE, the first 19 items are phrased to measure fears related to contamination and illnesses (e.g., “*I am afraid*….”). On the other hand, items 20–33 describe some behaviors that can be a consequence of those fears. However, even in the presence of low fears, individuals may score very high on the behaviors listed in these items, as a result, for example, of general recommendations to engage in virus-mitigating behaviors (e.g., “*I use purell/other sanitizer*,” “*I work or do my job on a computer*,” “*I wash my hands at times other than just using the bathroom or before eating*.”). This is why, as per the developer’s instructions, these items should not be considered toward scoring a general fear factor and likely provide ancillary information about the extent to which exposure to an illness or virus alters people’s behaviors (Ehrenreich-May and Saez, personal communication). Finally, the content of items 34 and 35 cover a general distress dimension (e.g., “*On average in the last week, being afraid of an illness or virus has caused me to experience strong emotions.”*) and a disturbance of the quality of life dimension (e.g., *“On average in the last week, being afraid of an illness or virus has gotten in the way of enjoying my life.”*). These are likely related constructs but not measures of fear themselves and thus can be equally considered to provide ancillary information about the exposure of an individual to an illness or virus situation such as a pandemic.

Based on this general description, we focused our content analysis on the first 19 fear items, which is indeed the main latent construct purportedly measured by the FIVE. A member of the research team with experience in qualitative studies and psychometric theory read the items and looked for alternative thematic organizations, extracting general categories representing the grouping of items. This was then confirmed by an independent rater following principles of open coding outlined by the grounded theory framework (Strauss and Corbin, 1998). Table 3 provides a visual representation of the proposed theoretical structure based on the content analysis as compared to the original structure. Four new subscales were thus extracted from this content analysis. The first category was named *fears of getting sick from an illness or virus*. The second subscale was called *fears that others may get sick from an illness or virus*. The third subscale was called *fears of concrete limitations due to an illness or virus*. The fourth subscale was called *fears of not being able to meet the basic needs of subsistence and work due to an illness or virus*.

**TABLE 3:** Provisional subscale and proposed subscale.

Item		Provisional subscale	Proposed subscale
1.	I may get a bad illness or virus.	Part 1. Fears about Contamination and Illness.	Fears of getting sick (ITC: 0.679)
2.	I will get very, very sick if I catch a bad illness or virus.	Part 1. Fears about Contamination and Illness.	Fears of getting sick (ITC: 0.793)
3.	I will have to go to the hospital because of a bad illness or virus	Part 1. Fears about Contamination and Illness.	Fears of getting sick (ITC: 0.712)
4.	I might die if I get a bad Illness or virus.	Part 1. Fears about Contamination and Illness.	Fears of getting sick (ITC: 0.742)
5.	My pet might get a bad illness or virus.	Part 1. Fears about Contamination and Illness.	Fears that others may get sick (ITC: 0.369)
6.	A family member might get sick or die because of a bad illness or virus	Part 1. Fears about Contamination and Illness.	Fears that others may get sick (ITC: 0.696)
7.	I may do something that would cause someone else to get a bad illness or virus.	Part 1. Fears about Contamination and Illness.	Fears that others may get sick (ITC: 0.512)
8.	A friend might get sick or die because of a bad illness or virus.	Part 1. Fears about Contamination and Illness.	Fears that others may get sick (ITC: 0.601)
9.	People in the world might get sick or die because of a bad illness or virus	Part 1. Fears about Contamination and Illness.	Fears that others may get sick (ITC: 0.377)
10.	I will be stuck at home because of a bad illness or virus.	Part 2. Fears about social distancing.	Fears of concrete limitations (ITC: 0.621)
11.	It will be hard to do things I like because of a bad illness or virus.	Part 2. Fears about social distancing.	Fears of concrete limitations (ITC: 0.721)
12.	I will miss a lot of work because of a bad illness or virus.	Part 2. Fears about social distancing.	Fears of not being able to meet the basic needs (ITC: 0.675)
13.	I will not be able to see friends (for a long time) because of a bad illness or virus	Part 2. Fears about social distancing.	Fears of concrete limitations (ITC: 0.656)
14.	I will do lose my job because of a bad illness or virus	Part 2. Fears about social distancing.	Fears of not being able to meet the basic needs (ITC: 0.535)
15.	I will lose my friends because of a bad illness or virus	Part 2. Fears about social distancing.	Fears of concrete limitations (ITC: 0.536)
16.	I will be sad and lonely because of a bad illness or virus	Part 2. Fears about social distancing.	Fears of concrete limitations (ITC: 0.662)
17.	I will not be able to celebrate good things (e.g., wedding, Birthday, etc.) because of a bad illness or virus	Part 2. Fears about social distancing.	Fears of concrete limitations (ITC: 0.625)
18.	I will not have enough food or supplies because of a bad illness or virus.	Part 2. Fears about social distancing.	Fears of not being able to meet the basic needs (ITC: 0.491)
19.	I will not have enough money to pay my bills or take care of my family because of a bad illness or virus	Part 2. Fears about social distancing.	Fears of not being able to meet the basic needs (ITC: 0.704)

*Item-total correlations (ITC) are presented in parentheses.*

We tested this newly proposed structure using a CFA procedure. The bottom part of Table 4 shows the results for these analyses. When we computed the new four-factor solution, the model fit improved significantly. Even though the fit for a bi-factor solution increases in the newly proposed item structure, the four-factor solution yields a better fit. The FIVE seems to behave as a multidimensional scale, measuring different components of the construct “fear of illness and virus.” We should thus interpret these results primarily using scores from the four subscales and not using an aggregated sum of fear items. It is important to note that, even though we found an acceptable fit, it was still below the criteria proposed by Hu and Bentler (1999). Because of this, we tried a final factor solution including only the first two factors. This is an important step if we consider that these are items that actually reflect to what extent individuals are afraid of infection, either for themselves or others, which we believe is the main underlying construct of the scale. For the proposed solution, we found an excellent fit (Table 4; two Fear Factors). We present factor loadings for the four and two-factor solutions in Table 2.

**TABLE 4:** Model fit indicators for examined factor structures (*n* = 159).

Model	CFI	TLI	RMSEA	SRMR
**Original factor structures proposed by developers**				
One overall factor	0.647	0.625	0.085	0.086
One fear factor (excludes the behaviors subscale)	0.694	0.655	0.129	0.099
Original theoretical four-factor solution	0.793	0.778	0.065	0.083
Bi-factor	0.836	0.816	0.059	0.067
**Proposed new factor structure based on content analysis**				
Content analysis: new four-factor solution	0.915	0.901	0.069	0.072
Content analysis: bi-factor solution.	0.911	0.886	0.075	0.061
Two fear factors	0.995	0.993	0.025	0.052

*CFI, Comparative Fit Index; TLI, Tucker–Lewis Fit Index; RMSEA, Root Mean Square Error of Approximation; SRMR, Standardized Root Mean Residual. The × 2 test of model fit was significant for every model.*

The original measure also included 14 items that measure different behaviors related to fear of illness and viruses. These items should not be used as indicators of fear of illness and viruses construct, as stated by the original authors (Ehrenreich-May and Saez, personal communication). We retained them as a supplement that may help assessors, both in clinical or in research settings, to quantify some behaviors related to illnesses and viruses. In the same way, the last two items provide an estimate on the overall level of impact of fear over two domains, emotionally and quality of life. These may be used as supplements, just like the behavior scale.

In short, we believe that only the first 19 items of the FIVE contribute to measuring a multidimensional construct, namely, *fear of illness and virus.* There is the long version with an acceptable fit consisting of four subscales, and the short one with an excellent fit focused on fears of self and others’ contagion. The remaining items constitute two supplemental sets of questions, namely, behaviors and impact ancillary information for the assessor, but not contributing to the measurement of the underlying construct.

Because of this, the next sections will provide information about the four subscales derived from our content analysis.

### Reliability

We examined the reliability of the newly proposed structures for the FIVE using Cronbach’s alpha (α), Composite reliability or Omega (ω; Viladrich et al., 2017), and average variance extracted (AVE; Fornell and Larcker, 1981), as the ratio of variance captured by the construct v/s error variance. All measures were calculated based on the structural model and using the “semTools” package for R (Jorgensen et al., 2019). All subscales exhibited an adequate degree of reliability by means of α and ω, while most of them had a ratio of explained variance above or equal to 50%:

The subscale *fears of getting sick from an illness or virus (FS)* showed α = 0.875, ω = 0.879, and AVE = 0.645; the subscale *fears that others may get sick from an illness or virus (FOS)* showed α = 0.744, ω = 0.762, and AVE = 0.402; the subscale fears of concrete limitations due to an illness or virus (FL) showed α = 0.854, ω = 0.857, and AVE = 0.503; while the subscale fears of not being able to meet basic needs of subsistence and work due to an illness or virus (FBN) showed α = 0.789, ω = 0.798, and AVE = 0.499. Finally, the overall Average Variance Extracted was 0.503.

Almost all average variance extracted values were above the higher correlation with the other constructs squared, an indication of discriminant validity (Fornell and Larcker, 1981). One exception was FOS, which showed a lower AVE than its squared correlation with FS (*r*^2^ = 0.461). In this case, it is expected not to have a high degree of discriminant validity as both latent variables refer to fear of contagion, differentiated by its target (oneself or others). We finally calculated the item-total correlations for each factor using the “multilevel” package for R (Bliese, 2016). Item-total correlations for the FS factor ranged from 0.679 to 0.793; for the FOS factor, they ranged from 0.369 to 0.696. The FL factor showed item-total correlations that ranged from 0.536 to 0.721, while they ranged from 0.491 to 0.704 in the FBN factor. Specific values can be seen in parentheses on Table 3.

### Test–Retest Reliability

Taking advantage of the longitudinal design of the study in which we evaluated the FIVE, we could examine the stability of the scores over time for each subscale. By design, the participants completed the FIVE every 7 days. We then calculated Pearson correlations between subscale scores of the first two applications, 7 days apart. We also calculated the intraclass correlation coefficient (ICC), given that both measures are widely used to test for stability and reliability. As there are many available models for ICCs, we opted for a 2-way mixed model with only one reading per occasion and based on absolute agreement, also called ICC_3_,_1_ (Trevethan, 2017), as it is considered an appropriate measure for repeated measurements. ICCs were calculated with the “icc3.inter.fn” function from the “irrICC” package for R (Gwet, 2019). Table 5 shows such correlations with ICC_3_,_1_’s in parentheses. All Pearson’s correlations were all statistically significant and strong, which is an indicator of adequate stability, together with equivalent scores of reliability by means of ICC.

**TABLE 5:** Test–retest Pearson correlations and ICC_3,1_ for absolute agreement between first and second assessment (7 days apart) for subscales and for total scale.

	FS—first assessment	FOS—first assessment	FL—first assessment	FBS—first assessment
FS—second assessment	0.724* (0.722)			
FOS—second assessment		0.804* (0.798)		
FL—second assessment			0.736* (0.727)	
FBS—second assessment				0.715* (0.715)

*p < 0.01; **FS**, fears of getting sick from an illness or virus; **FOS**, fears that significant others may get sick from an illness or virus; **FL**, ears of concrete limitations due to an illness or virus; FBN, fears of not being able to meet basic needs of subsistence and work due to an illness or virus. ICC_3,1_ is provided in parentheses.

### Convergent Validity

We examined the convergent validity of the FIVE with respect to depressive symptoms and PTSD. During the COVID-19 pandemic, it is reasonable to think that levels of fear of contamination and disease, fears of others becoming sick, fears of the limitations due to confinement, and fears of not meeting basic needs should correlate with indicators of traumatic reactivity and depression. This has been found in studies even during the COVID-19 pandemic such as the one carried out by Moreno et al. (2020) and by Tang et al. (2020).

As can be seen in Table 6, all subscales were significantly and positively correlated with both depressive symptomatology (PHQ-9) and posttraumatic stress (ITQ) at time point 1. This indicates that individuals who reported higher levels of fear also tended to report higher posttraumatic stress and depressive symptomatology.

**TABLE 6:** Concurrent validity correlation table.

Variable	*M*	*SD*	1	2	3	4	5
Fears of getting sick	8.28	2.93					
Fears that other can get sick	10.49	3.13	0.58**				
Fears of concrete limitations	11.59	4.24	0.48**	0.55**			
Fears of not being able to meet the basic needs	8.68	3.24	0.38**	0.39**	0.57**		
PHQ 1	10.95	5.77	0.33**	0.36**	0.51**	0.55**	
PTSD 1	8.43	5.11	0.39**	0.42**	0.45**	0.47**	0.50**

M and SD are used to represent mean and standard deviation, respectively. ** indicates p < 0.01. PHQ 1 = depressive symptomatology at time 1, PTSD 1 = posttraumatic symptomatology at time 1.

To further explore the predictive validity of the FIVE, we fitted a separate OLS regression for each subscale predicting depressive symptomatology (PHQ-9) 5 days after baseline, and posttraumatic stress symptomatology (ITQ) 7 days after baseline. All equations were controlled for initial PHQ-9 and ITQ values. Because there were eight equations, a Bonferroni correction was applied by dividing our critical alpha value of 0.05 by 8 (0.006) while interpreting the results to correct for type 1 error probability (Haynes, 2013). We present details of the main parameters in Table 7.

**TABLE 7:** Regression results predicting future posttraumatic stress and depressive symptomatology.

Predictors	Depressive symptoms			Posttraumatic symptoms		
	Beta	SE	*P*-value	Beta	SE	*P*-value
Fears of getting sick	0.133	0.089	0.138	0.428	0.131	0.001
Fears that other can get sick	0.129	0.087	0.138	0.371	0.129	0.005
Fears of concrete limitations	0.099	0.068	0.147	0.335	0.094	0.001
Fears of not being able to meet the basic needs	0.124	0.091	0.177	0.392	0.121	0.002

*Unstandardized beta coefficients are presented with their standard error and associated p-value. Each predictor was calculated by computing a different equation. All equations are controlled by baseline values for the criterion variable.*

None of the four subscales significantly predicted depressive symptoms later in time. However, when future posttraumatic stress symptoms were predicted and controlled by baseline values, fears of getting sick [*b* = 0.428, *t*(130) = 3.273, *p* = 0.001], fear that others get sick [*b* = 0.371, *t*(128) = 2.889, *p* = 0.005], fears related to limitations due to a virus or illness [*b* = 0.335, *t*(131) = 3.566, *p* = 0.001], and fears related to fears about not meeting the basic needs for subsistence or work [*b* = 0.392, *t*(131) = 3.234, *p* = 0.002] at time one exerted a significant effect, even when correcting for multiple comparisons. Altogether, these results indicate that people who are more scared of contagion and lockdown consequences tend to be more depressed and show more posttraumatic stress symptoms concurrently. These fears, however, only predict subsequent posttraumatic stress.

## Discussion

The study described in this article was part of a longitudinal study conducted for understanding the effects that quarantine and isolation may have on mental health during the COVID-19 pandemic. When deciding which variables to include, we repeatedly found fear and fear-related constructs as one of the most reported emotions in studies about the effects of quarantine and isolation because of a virus (Gu et al., 2015; Brooks et al., 2020; Lima et al., 2020; Montemurro, 2020; Moreno et al., 2020; Tang et al., 2020; Thakur and Jain, 2020). Measuring fear seemed to be a key component for understanding the effects of the COVID-19 pandemic on mental health as well as on behaviors that can contribute to either propagating or mitigating the infection (Ahorsu et al., 2020; Harper et al., 2020).

When facing the challenge of finding an instrument that could capture this construct profoundly enough to understand people’s emotional response to such a massive and pervasive phenomenon, we found that most of the existing instruments were either unidimensional or restricted to be used in the COVID-19 pandemic. In this context, we found the FIVE, which is both multidimensional and, even though developed during the COVID-19 pandemic, extendable to other pandemics or further life threatening situations.

The FIVE was originally proposed by Ehrenreich (2020), with a theoretical distribution of items measuring two facets of a fear factor, namely, *fears about contamination and illness* and *fears about social distancing*, not being exclusively applicable to the current COVID-19 pandemic. The authors included two additional scales, one for *behaviors related to fear of illness and virus* and one for the *impact of fear of illness and virus*. This structure was found to provide a bad fit to the data in a sample of 159 adult individuals during the COVID-19 pandemic in Chile. Content analysis revealed that items covering fear (e.g., “I am afraid…”) could be more aptly distributed in four groups covering facets of the fear of illness and virus construct.

As proposed by this psychometric study, our adaptation of the FIVE consists of a 19-item multidimensional scale that measures four components of the underlying *fear of illness and virus* construct. These components work as subscales of the instrument, namely, *fears of getting sick*, *fear that others will get sick*, *fears about the limitations due to an illness or a virus*, and *fears about not meeting the basic needs for subsistence or work*. We found an acceptable fit for the model including all four subscales, however, subthreshold based on criteria proposed by Hu and Bentler (1999). We found an excellent fit to the data on a smaller scale, including only the first two factors. All subscales showed equivalent subsequent psychometric properties, including good internal consistency, test–retest reliability, convergent validity, and predictive power. Based on this, we recommend the reader to use the four factors when a broader coverage of constructs is preferred, while using a short version composed of the first two subscales when construct validity is preferred, with the cost of restricting the measurement to fears of contagion to oneself or others.

Our adaptation also includes two supplemental scales that provide ancillary information about behaviors potentially associated with the fears, and to the extent to which fears may have led individuals to experience strong emotions and to decrease their quality of life. These supplemental scales do not represent the multidimensional *fear of illness and virus* construct but may be useful to understand other areas of affectation during a pandemic or exposure to illness. However, it is not clear if the behaviors listed in the first supplemental scale (i.e., behaviors related to fear of illness and virus) are in fact related to fears or can be a result of adaptive behaviors prescribed by health institutions or governments (e.g., “handwashing other than after using the bathroom or before eating”). The relationship between fears of illness and viruses, with behaviors, needs to be further examined. We thus recommend using the first 19 items (short format from 1 to 9, and long format from 1 to 19) to measure fears related to viruses and the consequences of lockdowns, items 20–33 if specific behaviors are of interest, and items 34 and 35 if affective consequences of the aforementioned fears are also a focus of interest.

We acknowledge that shorter measures have been developed to measure fear during the Covid-19 pandemic. However, the FIVE may have three distinct advantages.

First, multidimensionality is one of the greatest advantages of this scale. This feature can further contribute to understanding the role of different facets of the construct of fear as related to exposure to an illness or a virus. Fear of illness and viruses has been related to both prosocial, virus-mitigating behaviors as well as harmful and virus-propagation behaviors. Different facets, as measured by the FIVE, may be differentially related to these outcomes. For example, fear of becoming sick may be a driver of discrimination and prejudice (Ren et al., 2020) and can inhibit treatment-seeking behaviors when needed (Lazzerini et al., 2020). However, fear of COVID-19 can also be a driver for virus-mitigating behaviors (Harper et al., 2020). A study using the Fear of COVID-19 scale supported a two-factor structure that differentiated emotional fear reactions from symptomatic expressions of fear such as heart palpitations or sleep problems. The second factor was highly correlated with anxiety symptoms, which supports the applicability of exploring different facets of fear (Tzur Bitan et al., 2020). Thus, the multidimensional structure of the FIVE may allow grouping specific information about fear and thus more nuanced associations with other psychological and behavioral outcomes.

Secondly, we believe this may work as a broader measure of fear of illness and virus, so that it may potentially be used to study other contexts in which exposure to illness and virus may be related to mental and behavioral health as well as to societal issues. Despite the content of the FIVE as it actually is restricted to the COVID-19 scenario, we believe it may be adapted so that its use could be generalized for future scenarios when fear is related to similar sources. We believe that developing empirically informed assessment instruments for measuring emotions such as the FIVE may not only help us develop and adapt massive psychosocial strategies but also let us improve communication strategies for heightening adherence to recommendations by inviting people to behave in such a way that could collectively help mitigate the damage by preventing contagion and protect those who present the highest risk of dying or end with long-lasting or permanent sequelae. We found that the FIVE was also related to depressive and posttraumatic stress symptomatology when assessed concurrently, showing that relatively higher scores in fear appear together with the aforementioned symptoms. Also, higher values of the FIVE at baseline predicted posttraumatic stress symptomatology later in time, highlighting its practical value. This may have concrete implications if those fears are subject to change based on the diffusion of public policies and communication strategies, as they may also serve as a protective strategy for the stress-related consequences of a pandemic. Evoking fear has been used as an adherence strategy in response to public health emergencies, such as vaccination promotion or behaviors that mitigate contagion (see Taylor, 2019). However, fear (e.g., particularly extreme fear) may lead to decreased preventive behaviors and increased psychological distress particularly in high-risk populations such as individuals with mental health conditions, mainly through irrational thinking (Chang et al., 2020). Thus, instruments like the FIVE may be useful not only for the evaluation of the psychological impact of fears—as it has been shown in its association with complex symptomatology and behaviors—but also for designing and implementing public health policies either general or group-specific. One clear discussion of how measuring fear can help communities decrease mental health consequences can be seen on Lin et al. (2020). The authors found that when individuals received negative COVID-19 information from social media, fear was magnified with increased levels of psychological distress in both adults and children (e.g., the fear of COVID-19 was found to be a mediator in the relation between problematic social media use and distress/insomnia) (Chen I.-H. et al., 2020; Lin et al., 2020). A clear example on how to distinguish between at-risk populations from others that are not at risk can be seen in research from Lin et al. (2020) who identified that individuals with problematic use of social media were particularly exposed to these risks, while Chen I.-H. et al. (2020) identified that fear of COVID-19 did not seem to be serious in children.

Thirdly, the FIVE is a freely available measure that can be easily adapted globally. The FIVE scale and correction templates of both English and Spanish versions—and probably additional languages in the future—were developed and intended to be kept free, brief, and accessible.

These results should be critically evaluated given their limitations. First, our study used a convenience sampling strategy, with relatively small sample size, making the results not generalizable to the population, especially for underrepresented samples without access to a reliable internet connection. However, according to known population parameters in Chile, we attempted to correct the accompanying biases by using quota sampling procedures. Second, the time window of the study was short, meaning that only predictions of short-term consequences of fears were granted, and thus our results may not be extended to longer periods of time. Third, our relatively small sample size did not allow for the test of measurement invariance (Kline, 2016) or a cross-validation procedure. However, we think that the proposal of a new factor structure with a good fit to the data based on a content analysis of the items, together with the prospective predictive power of the FIVE with regard to post-traumatic stress symptomatology, is an encouraging result that requires further investigation by using larger sample sizes and longer time windows, together with a probabilistic design if a representation of the population is sought for.

Despite its limitations, our study provided the translation and evaluation of the psychometric properties of an instrument capable of measuring fears of getting sick and the consequences of social isolation and quarantine during a pandemic in a region badly affected by it. It is estimated that the COVID-19 pandemic is not an isolated event (e.g., Frutos et al., 2020) but a catastrophic event with a high likelihood of reappearing in the coming years after its resolution. We have also learned from previous pandemics that their consequences are usually prevalent and long-lasting in nature (Maunder et al., 2003; Taylor, 2019; Ornell et al., 2020), highlighting the need to measure them. Future studies are needed to confirm these findings, expand them to other mental health outcomes, and explain through which mechanisms they operate. These efforts may grant us more preparation for the mental health problems that will appear as a consequence of the pandemic and the needed public health policies, together with more preparation for future similar situations.

## Data Availability Statement

The raw data supporting the conclusions of this article will be made available by the authors, without undue reservation, to any qualified researcher.

## Ethics Statement

The studies involving human participants were reviewed and approved by the Committee of Ethics in Science of the Universidad Adolfo Ibáñez. The patients/participants provided their written informed consent to participate in this study.

## Author Contributions

MC contributed to the conception of the study, and to the conception, drafting, and editing process of the manuscript, and approved the final version. CH and AB contributed to the conception and design of the study, the collection and interpretation of data, and approved the final version. CN and NL contributed to the acquisition of the data, drafting of the manuscript, and approved the final version. YQ and AD contributed to the drafting process of the manuscript and approved the final version. All authors contributed to the article and approved the submitted version.

## Conflict of Interest

The authors declare that the research was conducted in the absence of any commercial or financial relationships that could be construed as a potential conflict of interest. The reviewer GM declared a shared affiliation with several of the authors, MC, YQ, to the handling editor at the time of review.
