# Supplementary material for: “What If We Get Sick?”: Spanish Adaptation and Validation of the Fear of Illness and Virus Evaluation Scale in a Non-clinical Sample Exposed to the COVID-19 Pandemic
Source: Front Psychol. 2021 Mar 11;12:590283. doi: 10.3389/fpsyg.2021.590283 (PMC7990903; doi:10.3389/fpsyg.2021.590283)
Supplement: Supplementary file 1 [file Data_Sheet_1.pdf]

Fear of Illness and Virus Evaluation (FIVE) – Adult Report Form

Rate how often you have felt afraid or worried about each item in the last week on the 1-4 scale below:

1. I am not afraid of this at all.
2. I am afraid of this some of the time.
3. I am afraid of this most of the time.
4. I am afraid of this all of the time.

Part 1. Fears about Contamination and Illness

	I am not afraid of this at all.	I am afraid of this some of the time.	I am afraid of this most of the time.	I am afraid of this all of the time.
1. I am afraid I may get a bad illness or virus.	1	2	3	4
2. I am afraid I will get very, very sick if I catch a bad illness or virus.	1	2	3	4
3. I am afraid I will have to go to the hospital because of a bad illness or virus.	1	2	3	4
4. I am afraid I might die if I get a bad illness or virus.	1	2	3	4
5. I am afraid my pet might get a bad illness or virus.	1	2	3	4
6. I am afraid a family member might get sick or die because of a bad illness or virus.	1	2	3	4
7. I am afraid I may do something that would cause someone else to get a bad illness or virus.	1	2	3	4
8. I am afraid a friend might get sick or die because of a bad illness or virus.	1	2	3	4
9. I am afraid people in the world might get sick or die because of a bad illness or virus.	1	2	3	4

Part 2. Fears about Social Distancing

	I am not afraid of this at all.	I am afraid of this some of the time.	I am afraid of this most of the time.	I am afraid of this all of the time.
10. I am afraid I will be stuck at home because of a bad illness or virus.	1	2	3	4
11. I am afraid it will be hard to do things I like because of a bad illness or virus.	1	2	3	4
12. I am afraid I will miss a lot of work because of a bad illness or virus.	1	2	3	4
13. I am afraid I will not be able to see friends (for a long time) because of a bad illness or virus.	1	2	3	4
14. I am afraid I will do lose my job because of a bad illness or virus.	1	2	3	4
15. I am afraid I will lose my friends because of a bad illness or virus.	1	2	3	4
16. I am afraid I will be sad and lonely because of a bad illness or virus.	1	2	3	4
17. I am afraid I will not be able to celebrate good things (e.g. wedding, birthday, etc.) because of a bad illness or virus.	1	2	3	4
18. I am afraid I will not have enough food or supplies because of a bad illness or virus.	1	2	3	4
19. I am afraid I will not have enough money to pay my bills or take care of my family because of a bad illness or virus.	1	2	3	4

<u>Now please rate how often you have done the things listed below in the last week on this 1-4 scale</u>:

1. I have not done this in last week.
2. I did this some of the time last week.
3. I did this most of the time last week.
4. I did this all the time last week.

Part 3. Behaviors Related to Illness and Virus Fears

	**I have not done this in last week.**	**I did this some of the time last week.**	**I did this most of the time last week.**	**I did this all the time last week.**
20. I stay away from people (other than those who live in my house).	1	2	3	4
21. I ask people if they are sick.	1	2	3	4
22. I avoid news or information about bad illnesses or viruses.	1	2	3	4
23. I wash my hands at times other than just after using the bathroom or before eating.	1	2	3	4
24. I wear a mask over my face or protective gear (e.g. gloves, things to cover my clothes).	1	2	3	4
25. I use Purell/other sanitizer.	1	2	3	4
26. I use Clorox/cleaners to wipe down surfaces.	1	2	3	4
27. I avoid touching things (e.g., phone, door knobs).	1	2	3	4
28. I avoid touching people (e.g., hugging, shaking hands).	1	2	3	4
29. I check the internet for illness or virus updates.	1	2	3	4
30. I use social media/FaceTime, etc. to stay connected to my friends.	1	2	3	4
31. I exercise outside.	1	2	3	4
32. I stay away from people inside my house (e.g., stay in another room or a certain distance away).	1	2	3	4
33. I work or do my job on a computer.	1	2	3	4

<u>For these next two items, please indicate how true the statement is you on the following scale</u>:

1. Not true for me at all.
2. Somewhat true.
3. Mostly true.
4. Definitely true.

Part 4. Impact of Illness and Virus Fears.

	**Not true for me at all.**	**Somewhat true.**	**Mostly true.**	**Definitely true.**
34. On average in the last week, being afraid of an illness or virus has caused me to feel experience strong emotions (e.g. anger, anxiety, sadness, irritable feelings, etc.).	1	2	3	4
35. On average in the last week, being afraid of an illness or virus has gotten in the way of enjoying my life (e.g. caused fights in my house, kept me from connecting with others, made me feel isolated or hopeless about the future, etc.).	1	2	3	4

The FIVE may be <u>freely</u> used with permission from Jill Ehrenreich-May, Ph.D. (j.ehrenreich@miami.edu). You are welcome to use this scale for your study, but please credit it appropriately as Ehrenreich-May (2020) in your work. We appreciate you sharing your findings with us too!
