# Supplementary material for: “What If We Get Sick?”: Spanish Adaptation and Validation of the Fear of Illness and Virus Evaluation Scale in a Non-clinical Sample Exposed to the COVID-19 Pandemic
Source: Front Psychol. 2021 Mar 11;12:590283. doi: 10.3389/fpsyg.2021.590283 (PMC7990903; doi:10.3389/fpsyg.2021.590283)
Supplement: Supplementary file 2 [file Table_1.docx]

**Evaluación del Miedo a la Enfermedad y al Virus, Validación Chilena (FIVE) –para Adultos**

Indique con cuanta frecuencia ha sentido miedo o preocupación sobre los siguientes ítems durante la última semana:

|  | **No estoy asustado o preocupado por esto en absoluto.** | **Estoy asustado o preocupado por esto algunas veces.** | **Estoy asustado o preocupado por esto la mayor parte del tiempo.** | **Estoy asustado o preocupado por esto todo el tiempo.** |
| --- | --- | --- | --- | --- |
| 1. Temo que podría contraer una enfermedad o un virus grave. | 1 | 2 | 3 | 4 |
| 2. Temo que me pondré muy, muy enfermo/a si contraigo una enfermedad o un virus. | 1 | 2 | 3 | 4 |
| 3. Temo que tendré que ir al hospital debido a una enfermedad o virus grave | 1 | 2 | 3 | 4 |
| 4. Temo que podría morir si contraigo una enfermedad o un virus grave. | 1 | 2 | 3 | 4 |
| 5. Temo que mi mascota podría contraer una enfermedad o un virus grave. | 1 | 2 | 3 | 4 |
| 6. Temo que un miembro de mi familia podría enfermarse o morir a causa de una enfermedad o virus grave. | 1 | 2 | 3 | 4 |
| 7. Temo que pueda hacer algo que cause que otra persona contraiga una enfermedad o un virus grave | 1 | 2 | 3 | 4 |
| 8. Temo que un amigo o amiga podría enfermarse o morir a causa de una enfermedad o virus grave. | 1 | 2 | 3 | 4 |
| 9. Temo que personas en el mundo podrían enfermarse o morir a causa de una enfermedad o virus grave | 1 | 2 | 3 | 4 |
| 10. Temo que me quedaré atrapado en casa debido a una enfermedad o virus grave. | 1 | 2 | 3 | 4 |
| 11. Temo que será difícil hacer cosas que me gustan debido a una enfermedad o virus grave | 1 | 2 | 3 | 4 |
| 12. Temo que perderé mucho trabajo debido a una enfermedad o virus grave | 1 | 2 | 3 | 4 |
| 13. Temo que no podré ver amigos (durante mucho tiempo) debido a una enfermedad o virus grave | 1 | 2 | 3 | 4 |
| 14. Temo que perderé mi trabajo debido a una enfermedad o virus grave | 1 | 2 | 3 | 4 |
| 15. Temo que perderé a mis amigos debido a una enfermedad o virus grave | 1 | 2 | 3 | 4 |
| 16. Temo que estaré triste y solo por una enfermedad o virus grave | 1 | 2 | 3 | 4 |
| 17. Temo que no podré celebrar cosas buenas (por ejemplo, bodas, cumpleaños, etc.) debido a una enfermedad o virus grave | 1 | 2 | 3 | 4 |
| 18. Temo que no tendré suficientes alimentos o suministros debido a una enfermedad o virus grave | 1 | 2 | 3 | 4 |
| 19. Temo que no tendré suficiente dinero para pagar mis cuentas o cuidar a mi familia debido a una enfermedad o virus grave | 1 | 2 | 3 | 4 |

Ahora, califique con qué frecuencia ha hecho las cosas enumeradas a continuación en la última semana:

|  | **No he hecho esto en la última**  **semana.** | **He hecho esto algunas veces en la última semana.** | **He hecho esto la mayor parte del tiempo en la última semana.** | **He hecho esto todo el tiempo en la última semana.** |
| --- | --- | --- | --- | --- |
| 20. Me mantengo alejado de las personas (que no sean las que viven en mi casa) | 1 | 2 | 3 | 4 |
| 21. Le pregunto a las personas si están enfermas o no | 1 | 2 | 3 | 4 |
| 22. Evito las noticias o información sobre enfermedades graves o virus. | 1 | 2 | 3 | 4 |
| 23. Me lavo las manos en momentos que no sean solo después de ir al baño o antes de comer | 1 | 2 | 3 | 4 |
| 24. Llevo una máscara sobre mi cara o equipo de protección (por ejemplo, guantes, cosas para cubrir mi ropa) | 1 | 2 | 3 | 4 |
| 25. Utilizo alcohol en gel u otro desinfectante | 1 | 2 | 3 | 4 |
| 26. Uso Cloro / o limpiadores para limpiar superficies | 1 | 2 | 3 | 4 |
| 27. Evito tocar las cosas (por ejemplo, el teléfono, puertas, control remoto). | 1 | 2 | 3 | 4 |
| 28. Evito tocar a las personas (por ejemplo, abrazar o dar la mano) | 1 | 2 | 3 | 4 |
| 29. Reviso internet para mantenerme informado/a sobre enfermedades o virus graves | 1 | 2 | 3 | 4 |
| 30. Uso las redes sociales (por ejemplo Facetime, Instagram, Facebook) para mantenerme en contacto con mis amigos y amigas | 1 | 2 | 3 | 4 |
| 31. Hago ejercicio afuera | 1 | 2 | 3 | 4 |
| 32. Me mantengo alejado de las personas dentro de mi casa (por ejemplo, me quedo en otra habitación o a cierta distancia) | 1 | 2 | 3 | 4 |
| 33. Trabajo o hago mi trabajo en una computadora | 1 | 2 | 3 | 4 |

Para las siguientes dos preguntas, indique cuán verdadera es la declaración sobre usted:

|  | **No es cierto para mí en absoluto.** | **Algo cierto.** | **La mayoría de las veces es cierto.** | **Totalmente cierto.** |
| --- | --- | --- | --- | --- |
| 34. En promedio, durante la última semana, tener miedo a una enfermedad o virus me ha provocado emociones fuertes (por ejemplo, ira, ansiedad, tristeza, irritabilidad) | 1 | 2 | 3 | 4 |
| 35. En promedio, durante la última semana, tener miedo de una enfermedad o un virus me ha impedido disfrutar de mi vida (por ejemplo, causó peleas en mi casa, me impidió conectarme con los demás, me hizo sentir aislado o desesperado sobre el futuro, etc. .) | 1 | 2 | 3 | 4 |
